# Supplementary material for: Aging measures and cancer in the Health and Retirement Study (HRS)
Source: Nat Commun. 2025 Jul 1;16:5916. doi: 10.1038/s41467-025-60913-z (PMC12215566; doi:10.1038/s41467-025-60913-z)
Supplement: Supplementary file 1 — Supplementary Information [file 41467_2025_60913_MOESM1_ESM.pdf]

Abbreviations:

Accel, age acceleration

BMI, body mass index

DNAm, DNA methylation

EC, epigenetic clock

HRS, Health and Retirement Study

KDM-BA, biological age metric estimated by the Klemera and Doubal method

mPOA, Dunedin methylation-pace of aging

PhenoAge, phenotypic age

SA, subjective age

SD, standard deviation

CMV, cytomegalovirus

HR, hazard ratio

OR, odds ratio

CI, confidence interval

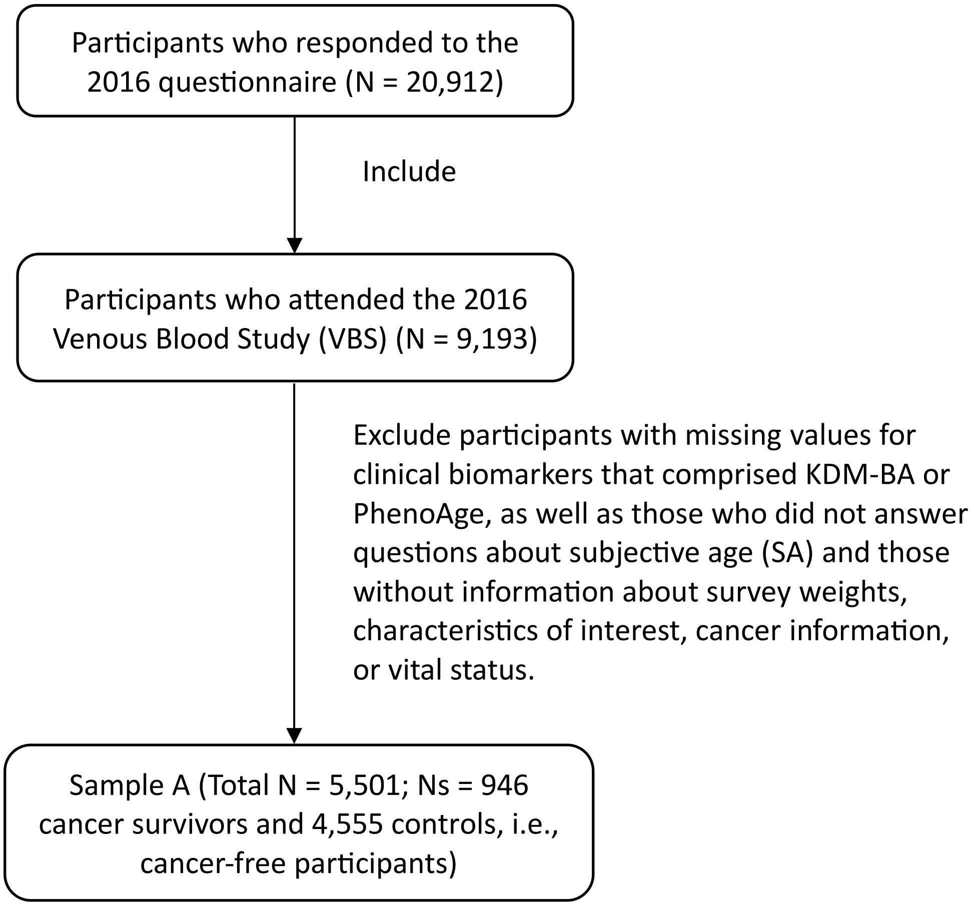

Supplementary Figure 1. Sample A

Note. Supplementary Figure 1 describes the inclusion and exclusion criteria for Sample A.

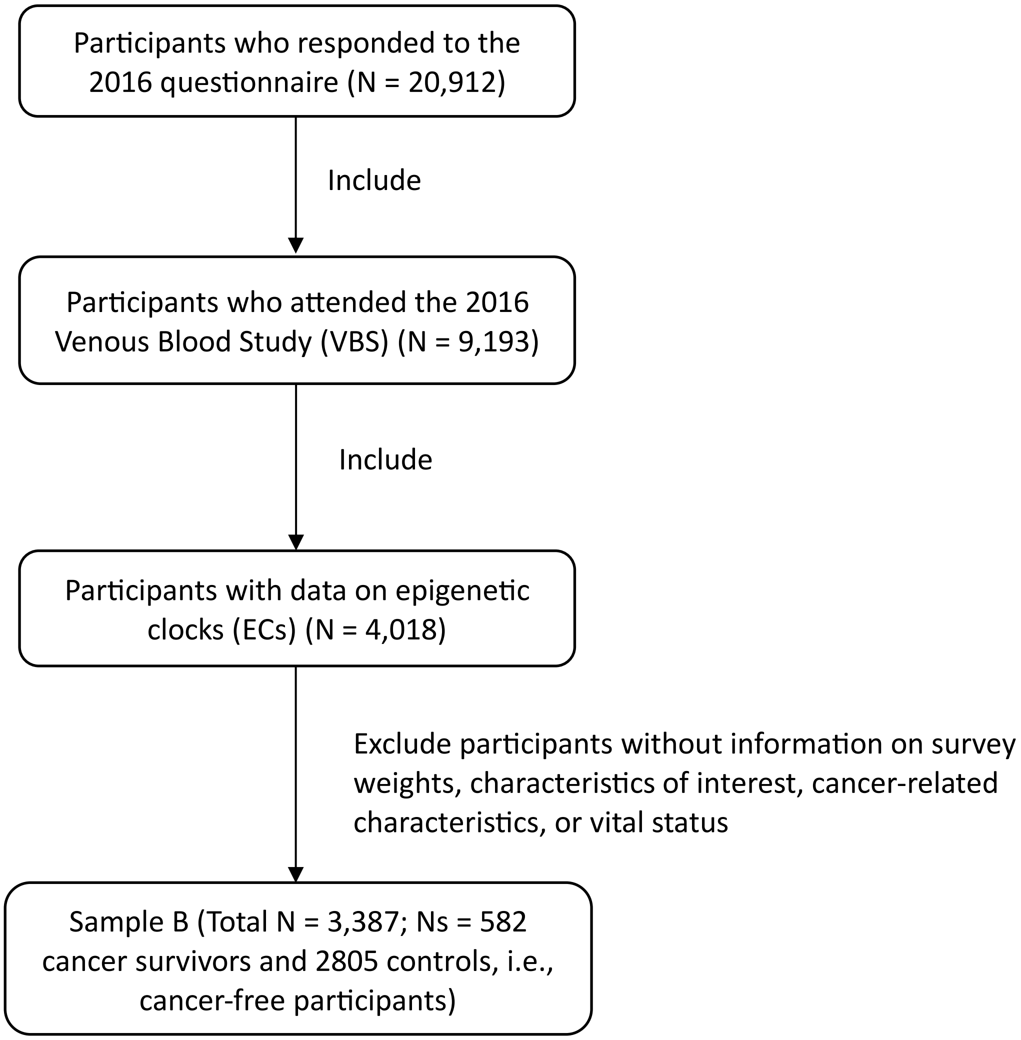

Supplementary Figure 2. Sample B

Note. Supplementary Figure 2 describes the inclusion and exclusion criteria for Sample B.

Supplementary Table 1. Distribution[a] of demographics and lifestyle factors among participants included and not included in the study with the 2016 Venous Blood Study (VBS) data for Sample A and with the DNA methylation data for Sample B; HRS

**Sample A**		
	Included in the study	Not included in the study
Mean chronological age, years (SD)	68.02 (0.25)	69.37 (0.37)
Male, %	46.0	45.7
Race/Ethnicity, %		
Non-Hispanic White	83.4	69.0
Non-Hispanic Black	7.4	14.3
Hispanic	4.3	8.2
Other	4.9	8.5
Education level, %		
< High School	10.6	20.8
High School	29.2	29.0
Greater than High School	60.2	50.2
Mean BMI, kg/m^2 (SD)	29.8 (0.10)	30.5 (0.19)
Smoking status, %		
Current smokers	9.9	12.9
Former smokers	44.1	43.7
**Sample B**		
	Included in the study	Not included in the study
Mean chronological age, years (SD)	68.5 (0.29)	68.6 (0.63)
Male, %	45.9	45.6
Race/Ethnicity, %		
Non-Hispanic White	78.4	72.0
Non-Hispanic Black	10.1	9.7
Hispanic	5.8	8.9
Other	5.7	9.4
Education level, %		
< High School	13.3	18.9
High School	29.4	33.1
Greater than High School	57.3	48
Mean BMI, kg/m^2 (SD)	29.9 (0.13)	29.9 (0.79)
Smoking status, %		
Current smokers	10.9	11.6
Former smokers	44.2	43.8

[a]Results were accounted for survey weights.

Supplementary Table 2. Cancer prevalence in 2016 in Sample A,[a] Sample B,[a] and the entire HRS cohort, as well as in Surveillance, Epidemiology, and End Results (SEER) (limited-duration prevalence) for cancers diagnosed in 1992-2016

Age groups	Sample A	Sample B	The entire HRS cohort	SEER
55-59	9.68%	9.30%	9.00%	5.78%
60-64	10.90%	10.50%	11.21%	8.39%
65-69	14.87%	15.86%	14.52%	12.20%
70-74	22.20%	20.76%	20.31%	16.71%
75-79	22.28%	20.64%	22.72%	20.10%
80-84	29.42%	23.46%	26.53%	22.11%
85+	24.18%	24.68%	26.66%	21.56%
All age combined	16.40%	15.74%	15.72%	13.21%

[a]Sample A included participants who reported their SA and had biomarker measures used to calculate KDM-BA and PhenoAge. Sample B included participants who had data on ECs.

Supplementary Table 3. Correlation of chronological age (CA) with aging constructs (KDM-BA, PhenoAge, and SA) and with age acceleration (Accel) for aging constructs in Sample A[a]

Correlation between CA and aging constructs				
	CA	KDM-BA	PhenoAge	SA
CA	1			
KDM-BA	0.92	1		
PhenoAge	0.80	0.84	1	
SA	0.60	0.56	0.53	1
**Correlation between CA and Accel for aging constructs**				
	CA	KDM-BA-Accel	PhenoAgeAccel	SA-Accel
CA	1			
KDM-BA-Accel	0	1		
PhenoAgeAccel	0	0.42	1	
SA-Accel	0	0.04	0.09	1

[a]Sample A included 5,501 participants who reported their SA and had biomarker measures used to calculate KDM-BA and PhenoAge.

Supplementary Table 4. Correlation of chronological age (CA) with epigenetic clocks (ECs) and with age acceleration (Accel) for ECs in Sample B[a]

Correlation between CA and ECs							
	CA	Hannum EC	Horvath EC	Levine EC	GrimAge	Zhang Score	mPOA
CA	1						
Hannum EC	0.81	1					
Horvath EC	0.73	0.77	1				
Levine EC	0.73	0.75	0.65	1			
GrimAge	0.83	0.76	0.65	0.74	1		
Zhang Score	0.31	0.49	0.28	0.47	0.54	1	
mPOA	0.03	0.12	0.10	0.20	0.38	0.36	1
**Correlation between CA and Accel for ECs**							
	CA	HannumAccel	HorvathAccel	LevineAccel	GrimAgeAccel	Zhang Score[b]	mPOA[c]
CA	1						
HannumAccel	0	1					
HorvathAccel	0	0.43	1				
LevineAccel	0	0.40	0.26	1			
GrimAgeAccel	0	0.25	0.10	0.34	1		
Zhang Score[b]	0.31	0.37	0.08	0.35	0.51	1	
mPOA[c]	0.03	0.16	0.12	0.25	0.63	0.36	1

[a]Sample B included 3,387 participants who had data on ECs.

[b]Consistent with previous publications, we did not calculate age acceleration for Zhang Score because it is a mortality risk score that was weakly correlated with CA.

[c]We did not calculate age acceleration for mPOA because it reflects the pace of the aging process and was not correlated with CA.

Supplementary Table 5. Mean (SD) of aging constructs among controls and cancer survivors with and without cancer treatments; HRS

Sample A[a]										
	**Controls**	**Cancer survivors**								
		Chemotherapy			Surgery			Radiation therapy		
		No	Yes	P-value[b]	No	Yes	P-value[b]	No	Yes	P-value[b]
KDM-BA-Accel, mean (SD)	-0.28 (0.06)	-0.13 (0.16)	-0.02 (0.33)	0.787	-0.08 (0.20)	-0.17 (0.20)	0.757	-0.09 (0.17)	-0.22 (0.31)	0.738
PhenoAgeAccel, mean (SD)	-0.60 (0.15)	-0.04 (0.28)	1.38 (0.58)	0.063	0.08 (0.35)	0.43 (0.45)	0.558	0.15 (0.28)	0.48 (0.73)	0.686
SA-Accel, mean (SD)	-0.27 (0.20)	0.82 (0.36)	2.36 (0.73)	0.114	1.59 (0.45)	0.18 (0.59)	0.091	1.34 (0.39)	-0.16 (0.86)	0.179

Sample B[a]										
	**Controls**	**Cancer survivors**								
		Chemotherapy			Surgery			Radiation therapy		
		No	Yes	P-value[b]	No	Yes	P-value[b]	No	Yes	P-value[b]
HannumAccel, mean (SD)	-0.12 (0.11)	1.07 (0.30)	0.76 (0.47)	0.648	1.14 (0.30)	0.78 (0.40)	0.479	0.98 (0.26)	1.22 (0.47)	0.715
HorvathAccel, mean (SD)	-0.04 (0.15)	0.53 (0.86)	1.94 (0.64)	0.124	0.58 (0.42)	1.15 (0.53)	0.423	0.79 (0.35)	0.67 (0.62)	0.885
LevineAccel, mean (SD)	-0.17 (0.20)	0.70 (0.36)	1.70 (0.51)	0.304	1.04 (0.43)	0.53 (0.40)	0.459	0.75 (0.32)	1.46 (0.26)	0.332
GrimAgeAccel, mean (SD)	-0.46 (0.12)	0.64 (0.26)	1.13 (0.37)	0.401	0.82 (0.30)	0.52 (0.34)	0.538	0.59 (0.23)	1.39 (0.58)	0.310
Zhang Score, mean (SD)	-1.15 (0.01)	-0.97 (0.02)	-0.94 (0.05)	0.576	-0.95 (0.03)	-1.00 (0.04)	0.391	-0.98 (0.02)	-0.87 (0.05)	0.057
mPOA, mean (SD)	-0.12 (0.11)	1.08 (0.005)	1.07 (0.01)	0.411	1.08 (0.005)	1.08 (0.01)	0.942	1.08 (0.005)	1.09 (0.01)	0.353

[a]Sample A included participants who reported their SA and had biomarker measures used to calculate KDM-BA and PhenoAge. Sample B included participants who had data on ECs.

[b]P-values were calculated using two-sided t-test based on the comparison across cancer treatment (Yes/No).

Supplementary Table 6. Associations between aging constructs and the prevalence of the most common individual cancers; HRS 2016

**Sample A[a]**			
	Numbers of prevalent cancer	OR (95% CI) per 1 SD increment in aging construct, p-value[b]	
		PhenoAgeAccel	SA-Accel
Prevalent breast cancer	204	0.94 (0.77, 1.16), p=0.568	0.97 (0.82, 1.14), p=0.667
Prevalent prostate cancer	253	**0.83 (0.70, 0.98), p=0.025**	1.04 (0.87, 1.24), p=0.638
Prevalent colorectal cancer	81	**1.24 (1.05, 1.47), p=0.012**	1.27 (0.98, 1.64), p=0.062
Prevalent lung cancer	44	**1.50 (1.11, 1.92), p=0.008**	1.01 (0.74, 1.38), p=0.929
**Sample B[a]**			
	Numbers of prevalent cancer	OR (95% CI) per 1 SD increment in aging construct, p-value[b]	
		HannumAccel	HorvathAccel
Prevalent breast cancer	122	1.17 (0.95, 1.43), p=0.138	1.10 (0.86, 1.41), p=0.433
Prevalent prostate cancer	149	0.87 (0.67, 1.13), p=0.304	0.87 (0.74, 1.02), p=0.091
Prevalent colorectal cancer	53	1.22 (0.98, 1.51), p=0.066	1.12 (0.71, 1.77), p=0.615
Prevalent lung cancer	29	1.24 (0.76, 2.03), p=0.385	1.41 (0.95, 2.09), p=0.087
	Numbers of prevalent cancer	OR (95% CI) per 1 SD increment in aging construct, p-value[b]	
		LevineAccel	GrimAgeAccel
Prevalent breast cancer	122	1.05 (0.83, 1.32), p=0.654	**1.27 (1.03, 1.67), p=0.029**
Prevalent prostate cancer	149	1.06 (0.88, 1.28), p=0.576	0.94 (0.74, 1.18), p=0.569
Prevalent colorectal cancer	53	1.13 (0.89, 1.43), p=0.321	**1.62 (1.10, 2.37), p=0.015**
Prevalent lung cancer	29	1.23 (0.88, 1.73), p=0.227	**2.94 (1.57, 5.50), p=0.001**
	Numbers of prevalent cancer	OR (95% CI) per 1 SD increment in aging construct, p-value[b]	
		Zhang Score	mPOA
Prevalent breast cancer	122	1.26 (0.95, 1.65), p=0.103	1.17 (0.90, 1.52), p=0.233
Prevalent prostate cancer	149	1.13 (0.86, 1.50), p=0.367	0.95 (0.69, 1.32), p=0.749
Prevalent colorectal cancer	53	1.25 (0.89, 1.76), p=0.192	1.22 (0.87, 1.72), p=0.252
Prevalent lung cancer	29	1.27 (0.57, 2.84), p=0.542	**1.86 (1.22, 2.85), p=0.005**

[a]Sample A included cancer survivors who reported SA and had biomarker measures used to calculate PhenoAge. Sample B included cancer survivors who had data on ECs.

[b]P-values were calculated using multivariable logistic regression model. The model was adjusted for chronological age, sex, race/ethnicity, education, BMI, smoking status, ever drinking, physical activity, comorbidity index, and CMV infection. In the analysis of ECs, the model was additionally adjusted for monocyte percentage, neutrophil percentage, and lymphocyte percentage. All analyses were accounted for survey weights.

Supplementary Table 7. Associations between aging constructs and mortality in cancer survivors stratified by sex; HRS (2016-2020)

Sample A[a]: 946 cancer survivors			
	HR (95% CI) per 1 SD increase in aging construct, p-value[b]		
Sex	KDM-BA-Accel	PhenoAgeAccel	SA-Accel
Female (59 deaths out of 511 survivors)	1.05 (0.80, 1.37), p=0.718	1.35 (1.01, 1.82), p=0.049	0.80 (0.52, 1.22), p=0.291
Male (63 deaths out of 435 survivors)	1.03 (0.72, 1.47), p=0.885	1.51 (0.90, 2.52), p=0.115	0.81 (0.54, 1.21), p=0.288
P-interaction[c]	0.614	0.894	0.875
**Sample B[a]: 582 cancer survivors**			
	HR (95% CI) per 1 SD increase in aging construct, p-value[b]		
Sex	HannumAccel	HorvathAccel	LevineAccel
Female (48 deaths out of 314 survivors)	2.18 (1.56, 3.04), p<0.001	1.35 (0.83, 2.21), p=0.221	1.61 (1.22, 2.12), p=0.001
Male (55 deaths out of 268 survivors)	1.04 (0.80, 1.36), p=0.749	0.96 (0.69, 1.36), p=0.831	1.51 (1.10, 2.07), p=0.012
P-interaction[c]	**0.005**	0.298	0.417
Sex	GrimAgeAccel	Zhang Score	mPOA
Female (48 deaths out of 314 survivors)	2.13 (1.53, 2.95), p<0.001	2.71 (1.94, 3.78), p<0.001	1.09 (0.67, 1.75), p=0.733
Male (55 deaths out of 268 survivors)	1.49 (1.07, 2.08), p=0.021	1.29 (0.92, 1.82), p=0.134	1.09 (0.76, 1.56), p=0.647
P-interaction[c]	**0.002**	**0.001**	0.050

[a]Sample A included cancer survivors who reported their SA and had biomarker measures used to calculate KDM-BA and PhenoAge. Sample B included cancer survivors who had data on ECs.

[b]P-values were calculated using Cox proportional hazards regression model. The model was adjusted for chronological age, race/ethnicity, education, BMI, smoking status, ever drinking, physical activity, comorbidity index, and CMV infection. In the analysis of ECs, the model was additionally adjusted for monocyte percentage, neutrophil percentage, and lymphocyte percentage. All analyses were accounted for survey weights.

[c]P-interaction was calculated by including an interaction term between sex and aging construct into the model.

Supplementary Table 8. Associations between aging constructs and mortality in cancer survivors stratified by race/ethnicity (among non-Hispanic White and non-Hispanic Black participants)[a]; HRS (2016-2020)

Sample A[b]: 946 cancer survivors			
	HR (95% CI) per 1 SD increase in aging construct, p-value[c]		
Race/Ethnicity	KDM-BA-Accel	PhenoAgeAccel	SA-Accel
Non-Hispanic White (107 deaths out of 775 survivors)	1.01 (0.82, 1.224), p=0.935	1.37 (1.03, 1.82), p=0.032	0.80 (0.55, 1.16), p=0.226
Non-Hispanic Black (9 deaths out of 93 survivors)	1.61 (0.93, 2.78), p=0.084	1.65 (0.63, 4.32), p=0.276	1.69 (0.71, 4.05), p=0.209
P-interaction[d]	0.691	**0.018**	0.548
Sample B[b]: 582 cancer survivors			
	HR (95% CI) per 1 SD increase in aging construct, p-value[c]		
Race/Ethnicity	HannumAccel	HorvathAccel	LevineAccel
Non-Hispanic White (83 deaths out of 449 survivors)	1.33 (1.07, 1.66), p=0.012	1.13 (0.83, 1.54), p=0.413	1.47 (1.15, 1.89), p=0.003
Non-Hispanic Black (15 deaths out of 75 survivors)	0.74 (0.43, 1.28), p=0.261	0.64 (0.49, 1.73), p=0.629	1.38 (0.64, 2.97), p=0.375
P-interaction[d]	0.973	0.635	0.247
Race/Ethnicity	GrimAgeAccel	Zhang Score	mPOA
Non-Hispanic White (83 deaths out of 449 survivors)	1.99 (1.47, 2.71), p<0.001	1.47 (1.14, 1.89), p=0.004	1.13 (0.80, 1.58), p=0.489
Non-Hispanic Black (15 deaths out of 75 survivors)	0.57 (0.33, 1.02), p=0.057	2.71 (0.72, 10.21), p=0.129	1.03 (0.26, 4.05), p=0.963
P-interaction[d]	0.154	0.974	0.711

[a]We only examined the associations among non-Hispanic White and Black participants due to a limited number of deaths among Hispanic participants and participants from other race groups.
[b]Sample A included cancer survivors who reported their SA and had biomarker measures used to calculate KDM-BA and PhenoAge. Sample B included cancer survivors who had data on ECs.
[c]P-values were calculated using Cox proportional hazards regression model. The model was adjusted for chronological age, sex, education, BMI, smoking status, ever drinking, physical activity, comorbidity index, and CMV infection. In the analysis of ECs, the model was additionally adjusted for monocyte percentage, neutrophil percentage, and lymphocyte percentage. All analyses were accounted for survey weights.
[d]P-interaction was calculated by including an interaction term between race/ethnicity and aging construct into the model.

Supplementary Table 9. Associations between aging constructs and mortality in cancer survivors stratified by chemotherapy; HRS (2016-2020)

Chemotherapy	KDM-BA-Accel	PhenoAgeAccel	SA-Accel
**Sample A[a]: 946 cancer survivors**			
	HR (95% CI) per 1 SD increase in aging construct, p-value[b]		
Yes (29 deaths out of 161 survivors)	0.98 (0.69, 1.39), p=0.896	2.12 (1.51, 2.99), p<0.001	1.47 (1.04, 2.06), p=0.029
No (93 deaths out of 785 survivors)	1.07 (0.84, 1.37), p=0.576	1.23 (0.97, 1.55), p=0.089	0.67 (0.48, 0.93), p=0.017
P-interaction[c]	0.738	0.060	**0.015**
**Sample B[a]: 582 cancer survivors**			
	HR (95% CI) per 1 SD increase in aging construct, p-value[b]		
Chemotherapy	HannumAccel	HorvathAccel	LevineAccel
Yes (24 deaths out of 88 survivors)	1.39 (0.86, 2.21), p=0.163	1.01 (0.63, 1.63), p=0.973	2.71 (1.89, 3.87), p<0.001
No (79 deaths out of 494 survivors)	1.34 (1.09, 1.67), p=0.006	1.24 (0.94, 1.65), p=0.116	1.67 (1.10, 1.97), p=0.001
P-interaction[c]	0.421	0.303	0.195
Chemotherapy	GrimAgeAccel	Zhang score	mPOA
Yes (24 deaths out of 88 survivors)	2.33 (1.51, 3.58), p<0.001	2.19 (1.39, 3.45), p=0.003	1.37 (0.79, 2.37), p=0.230
No (79 deaths out of 494 survivors)	1.69 (1.22, 2.35), p=0.002	1.48 (1.13, 1.94), p=0.005	1.08 (0.78, 1.50), p=0.632
P-interaction[c]	0.220	0.373	0.453

[a]Sample A included cancer survivors who reported their SA and had biomarker measures used to calculate KDM-BA and PhenoAge. Sample B included cancer survivors who had data on ECs.

[b]P-values were calculated using Cox proportional hazards regression model. The model was adjusted for chronological age, sex, race/ethnicity, education, BMI, smoking status, ever drinking, physical activity, comorbidity index, as well as CMV infection. In the analysis of ECs, the model was additionally adjusted for monocyte percentage, neutrophil percentage, and lymphocyte percentage. All analyses were accounted for survey weights.

[c]P-interaction was calculated by including an interaction term between chemotherapy and aging construct into the model.

Supplementary Table 10. Associations between aging constructs and mortality in cancer survivors stratified by surgery; HRS (2016-2020)

Sample A[a]: 946 cancer survivors			
	HR (95% CI) per 1 SD increase in aging construct, p-value[b]		
Surgery	KDM-BA-Accel	PhenoAgeAccel	SA-Accel
Yes (49 deaths out of 346 survivors)	1.08 (0.75, 1.56), p=0.683	1.58 (0.97, 2.56), p=0.064	0.75 (0.51, 1.10), p=0.134
No (73 deaths out of 600 survivors)	1.02 (0.78, 1.33), p=0.908	1.28 (1.96, 1.70), p=0.092	0.87 (0.56, 1.35), p=0.527
P-interaction[c]	0.548	0.289	0.425
**Sample B[a]: 582 cancer survivors**			
	HR (95% CI) per 1 SD increase in aging construct, p-value[b]		
Surgery	HannumAccel	HorvathAccel	LevineAccel
Yes (29 deaths out of 184 survivors)	1.59 (1.10, 2.29), p=0.015	1.32 (0.79, 2.22), p=0.274	2.09 (1.27, 3.45), p=0.005
No (74 deaths out of 398 survivors)	1.11 (0.78, 1.57), p=0.565	1.10 (0.79, 1.53), p=0.576	1.51 (1.15, 1.99), p=0.004
P-interaction[c]	0.173	0.244	0.411
Surgery	GrimAgeAccel	Zhang Score	mPOA
Yes (29 deaths out of 184 survivors)	2.27 (1.16, 4.42), p=0.018	1.49 (0.94, 2.34), p=0.086	1.25 (0.61, 2.56), p=0.522
No (74 deaths out of 398 survivors)	1.72 (1.22, 2.42), p=0.003	1.74 (1.33, 2.27), p<0.001	1.15 (0.81, 1.61), p=0.428
P-interaction[c]	0.244	0.797	0.356

[a]Sample A included cancer survivors who reported their SA and had biomarker measures used to calculate KDM-BA and PhenoAge. Sample B included cancer survivors who had data on ECs.

[b]P-values were calculated using Cox proportional hazards regression model. The model was adjusted for chronological age, sex, race/ethnicity, education, BMI, smoking status, ever drinking, physical activity, comorbidity index, as well as CMV infection. In the analysis of ECs, the model was additionally adjusted for monocyte percentage, neutrophil percentage, and lymphocyte percentage. All analyses were accounted for survey weights.

[c]P-interaction was calculated by including an interaction term between surgery and aging construct into the model.

Supplementary Table 11. Associations between aging constructs and mortality in cancer survivors stratified by radiation therapy; HRS (2016-2020)

Sample A[a]: 946 cancer survivors			
	HR (95% CI) per 1 SD increase in aging construct, p-value[b]		
Radiation therapy	KDM-BA-Accel	PhenoAgeAccel	SA-Accel
Yes (26 deaths out of 165 survivors)	1.33 (0.74, 2.38), p=0.326	1.70 (0.94, 3.07), p=0.079	0.89 (0.56, 1.45), p=0.651
No (96 deaths out of 781 survivors)	1.00 (0.78, 1.29), p=0.985	1.34 (1.01, 1.78), p=0.044	0.81 (0.60, 1.17), p=0.262
P-interaction[c]	0.694	0.918	0.781
**Sample B[a]: 582 cancer survivors**			
	HR (95% CI) per 1 SD increase in aging construct, p-value[b]		
Radiation therapy	HannumAccel	HorvathAccel	LevineAccel
Yes (20 deaths out of 92 survivors)	1.30 (0.62, 2.71), p=0.453	1.27 (0.98, 164), p=0.064	1.94 (1.01, 3.73), p=0.047
No (83 deaths out of 490 survivors)	1.35 (1.08, 1.69), p=0.009	1.25 (0.93, 1.68), p=0.137	1.45 (1.11, 1.87), p=0.006
P-interaction[c]	0.909	0.871	0.727
Radiation therapy	GrimAgeAccel	Zhang Score	mPOA
Yes (20 deaths out of 92 survivors)	2.42 (1.00, 5.89), p=0.050	3.48 (0.80, 15.22), p=0.090	0.65 (0.31, 1.36), p=0.224
No (83 deaths out of 490 survivors)	1.83 (1.34, 2.48), p<0.001	1.58 (1.20, 2.07), p=0.001	1.23 (0.87, 1.73), p=0.241
P-interaction[c]	0.406	0.735	0.148

[a]Sample A included cancer survivors who reported their SA and had biomarker measures used to calculate KDM-BA and PhenoAge. Sample B included cancer survivors who had data on ECs.

[b]P-values were calculated using Cox proportional hazards regression model. The model was adjusted for chronological age, sex, race/ethnicity, education, BMI, smoking status, ever drinking, physical activity, comorbidity index, as well as CMV infection. In the analysis of ECs, the model was additionally adjusted for monocyte percentage, neutrophil percentage, and lymphocyte percentage. All analyses were accounted for survey weights.

[c]P-interaction was calculated by including an interaction term between radiation therapy and aging construct into the model.

Supplementary Table 12. Associations between aging constructs and mortality in cancer survivors stratified by time since cancer diagnosis; HRS (2016-2020)

Sample A: 941 cancer survivors (5 survivors were excluded due to missing time of cancer diagnosis)			
	HR (95% CI) per 1 SD increase in aging construct, p-value[b]		
Time since cancer diagnosis	KDM-BA-Accel	PhenoAgeAccel	SA-Accel
<2 years (14 deaths out of 95 survivors)	1.06 (0.56, 2.04), p=0.841	1.42 (0.71, 2.85), p=0.298	1.53(0.59, 3.98), p=0.359
2-5 years (25 deaths out of 162 survivors)	0.89 (0.52, 1.52), p=0.663	1.85 (0.78, 4.35), p=0.155	0.80 (0.44, 1.45), p=0.452
>5 years (83 deaths out of 683 survivors)	1.02 (0.81, 1.28), p=0.851	1.26 (0.89, 1.77), p=0.190	0.81 (0.56, 1.16), p=0.246

Sample B: 577 cancer survivors (5 survivors were excluded due to missing time of cancer diagnosis)			
	HR (95% CI) per 1 SD increase in aging construct, p-value[b]		
Time since cancer diagnosis	HannumAccel	HorvathAccel	LevineAccel
<2 years (15 deaths out of 64 survivors)	1.18 (0.70, 2.00), p=0.486	1.67 (0.37, 7.54), p=0.450	23.15 (8.17, 65.61), p<0.001
2-5 years (15 deaths out of 110 survivors)	1.17 (0.68, 2.01), p=0.564	1.92 (1.35, 2.73), p<0.001	2.92 (0.95, 9.01), p=0.061
>5 years (73 deaths out of 403 survivors)	1.35 (1.14, 1.76), p=0.023	1.17 (0.89, 1.55), p=0.262	1.42 (1.14, 1.77), p=0.002
Time since cancer diagnosis	GrimAgeAccel	Zhang Score	mPOA
<2 years (15 deaths out of 64 survivors)	1.45 (1.18, 1.78), p=0.003	2.33 (1.87, 2.88), p<0.001	0.92 (0.45, 1.88), p=0.790
2-5 years (15 deaths out of 110 survivors)	4.57 (2.13, 9.79), p<0.001	1.03 (0.50, 2.12), p=0.931	2.89 (0.47, 9.28), p=0.316
>5 years (73 deaths out of 403 survivors)	1.80 (1.26, 2.57), p=0.002	1.52 (1.15, 2.02), p=0.004	1.12 (0.77, 1.61), p=0.552

[a]Sample A included cancer survivors who reported their SA and had biomarker measures used to calculate KDM-BA and PhenoAge. Sample B included cancer survivors who had data on ECs.

[b]P-values were calculated using Cox proportional hazards regression model. The model was adjusted for chronological age, sex, race/ethnicity, education, BMI, smoking status, ever drinking, physical activity, comorbidity index, as well as CMV infection. In the analysis of ECs, the model was additionally adjusted for monocyte percentage, neutrophil percentage, and lymphocyte percentage. All analyses were accounted for survey weights.

Supplementary Table 13. Associations between aging constructs and cancer prevalence in 2016; cancer survivors whose cancer was diagnosed within two years of blood collection were excluded; HRS

Sample A[a]				
Aging constructs	No. of cancer survivors	No. of controls	OR (95% CI) per 1 SD increase in aging construct, p-value[b]	
			Model 1[c]	Model 2[c]
KDM-BA-Accel (SD = 3.81 years)			1.05 (0.95, 1.15), p=0.289	1.00 (0.90, 1.11), p=0.994
PhenoAgeAccel (SD = 7.11 years)	845	4,555	**1.14 (1.04, 1.25), p=0.003**	1.08 (0.97, 1.18), p=0.217
SA-Accel (SD = 10.37 years)			**1.17 (1.08, 1.28), p<0.001**	1.13 (1.04, 1.24), p=0.007
Sample B[a]				
Aging constructs	No. of cancer survivors	No. of controls	OR (95% CI) per 1 SD increase in aging construct, p-value[b]	
			Model 1[b]	Model 2[c]
HannumAccel (SD = 5.20 years)			**1.23 (1.08, 1.39), p=0.002**	**1.22 (1.08, 1.37), p=0.002**
HorvathAccel (SD = 6.38 years)			1.13 (0.99, 1.31), p=0.076	1.12 (0.98, 1.28), p=0.109
LevineAccel (SD = 6.74 years)			**1.15 (1.02, 1.28), p=0.027**	1.13 (1.00, 1.27), p=0.050
GrimAgeAccel (SD = 4.65 years)	513	2,805	**1.23 (1.07, 1.41), p=0.003**	**1.20 (1.03, 1.40), p=0.025**
Zhang Score (SD = 0.45 units)			**1.34 (1.15, 1.55), p<0.001**	**1.29 (1.10, 1.53), p=0.002**
POA (SD = 0.09 years of physiological decline per one chronological year)			1.14 (0.98, 1.31), p=0.090	1.08 (0.94, 1.25), p=0.279

[a]Sample A included participants who reported their SA and had biomarker measures used to calculate KDM-BA and PhenoAge. Sample B included participants who data on ECs.

[b]P-values were calculated from multivariable logistic regression.

[c]Model 1 was adjusted for chronological age, sex, and race/ethnicity. Model 2 was additionally adjusted for BMI, smoking status, ever drinking, physical activity, comorbidity index, as well as CMV infection. In the analysis of ECs, both model were additionally adjusted for monocyte percentage, neutrophil percentage, and lymphocyte percentage. All analyses were accounted for survey weights.

Supplementary Table 14. Associations between aging constructs and mortality in cancer survivors; cancer survivors whose cancer was diagnosed within two years of blood collection were excluded; HRS (2016-2020)

Sample A[a]				
Cancer survivors (N = 845)				
Aging constructs	No. of deaths	Total person-year	HR (95% CI) per 1 SD increase in aging construct, p-value[b]	
			Model 1[c]	Model 2[c]
KDM-BA-Accel (SD = 4.02 years)	108	3,379	1.15 (0.96, 1.37), p=0.136	1.00 (0.81, 1.23), p=0.961
PhenoAgeAccel (SD = 7.19 years)			**1.47 (1.12, 1.92), p=0.006**	1.33 (1.00, 1.82), p=0.050
SA-Accel (SD = 10.33 years)			0.99 (0.68, 1.43), p=0.933	0.83 (0.58, 1.18), p=0.289
Sample B[a]				
Cancer survivors (N = 513)				
Aging constructs	No. of deaths	Total person-year	HR (95% CI) per 1 SD increase in aging construct, p-value[b]	
			Model 1[c]	Model 2[c]
HannumAccel (SD = 5.48 years)	88	2,011	**1.41 (1.07, 1.86), p=0.015**	1.33 (1.00, 1.78), p=0.050
HorvathAccel (SD = 6.94 years)			1.22 (0.93, 1.59), p=0.147	1.21 (0.94, 1.57), p=0.141
LevineAccel (DNAmPhenoAge) (SD = 6.89 years)			**1.52 (1.23, 1.87), p<0.001**	**1.49 (1.20, 1.86), p<0.001**
GrimAgeAccel (SD = 4.66 years)			**2.08 (1.55, 2.77), p<0.001**	**1.86 (1.34, 2.57), p<0.001**
Zhang Score (SD = 0.46 units)			**1.64 (1.23, 2.19), p=0.001**	1.51 (1.13, 2.01), p=0.006
mPOA (SD = 0.09 years of physiological decline per one chronological year)			1.20 (0.90, 1.59), p=0.208	1.06 (0.76, 1.48), p=0.710

[a]Sample A included participants who reported their SA and had biomarker measures used to calculate KDM-BA and PhenoAge. Sample B included participants who had data on ECs.

[b]P-values were calculated from multivariable Cox proportional hazards regression.

[c]Model 1 was adjusted for chronological age, sex, and race/ethnicity. Model 2 was additionally adjusted for BMI, smoking status, ever drinking, physical activity, comorbidity index, as well as CMV infection. In the analysis of ECs, both models were additionally adjusted for monocyte percentage, neutrophil percentage, and lymphocyte percentage. All analyses were accounted for survey weights.

Supplementary Table 15. Associations between aging constructs and risk of any type cancer; HRS (2016-2020)

Aging constructs	No. of cancer cases	Total person-years	HR (95% CI) per 1 SD increase in aging construct, p-value[b]	
Sample A: 4,413 participants without a history of cancer in 2016[a]				
			Model 1[c]	Model 2[c]
KDM-BA-Accel (SD = 3.76 years)	182	17,405	1.04 (0.88, 1.23), p=0.638	1.01 (0.84, 1.20), p=0.955
PhenoAgeAccel (SD = 7.07 years)			1.05 (0.86, 1.29), p=0.637	0.94 (0.76, 1.18), p=0.594
SA-Accel (SD = 10.35 years)			1.00 (0.82, 1.22), p=0.962	0.96 (0.81, 1.15), p=0.679
Sample B: 2,688 participants without a history of cancer in 2016[a]				
Aging constructs	No. of cancer cases	Total person-years	HR (95% CI) per 1 SD increase in aging construct, p-value[b]	
			Model 1[c]	Model 2[c]
HannumAccel (SD = 5.13 years)	122	10,366	1.15 (0.93, 1.42), p=0.187	1.14 (0.92, 1.41), p=0.232
HorvathAccel (SD = 6.26 years)			1.06 (0.88, 1.29), p=0.521	1.06 (0.88, 1.27), p=0.540
LevineAccel (SD = 6.77 years)			1.07 (0.83, 1.38), p=0.611	1.07 (0.83, 1.37), p=0.588
GrimAgeAccel (SD = 4.64 years)			1.24 (0.94, 1.64), p=0.127	1.17 (0.85, 1.62), p=0.331
Zhang Score (SD = 0.45 units)			1.00 (0.74, 1.35), p=0.997	0.97 (0.72, 1.32), p=0.873
mPOA (SD = 0.09 years of physiological decline per one chronological year)			1.29 (0.93, 1.78), p=0.131	1.20 (0.83, 1.74), p=0.323

[a]Sample A included 4,413 participants who reported their SA and had biomarker measures used to calculate KDM-BA and PhenoAge. Sample B included 2,688 participants who had data on ECs. Participants with a prevalent cancer in 2016 and with missing cancer status after 2016 or missing cancer diagnosis time were excluded.

[b]P-values were calculated from multivariable Cox proportional hazards regression.

[c]Model 1 was adjusted for chronological age, sex, and race/ethnicity. Model 2 was additionally adjusted for BMI, smoking status, ever drinking, physical activity, comorbidity index, as well as CMV infection. In the analysis of ECs, both models were additionally adjusted for monocyte percentage, neutrophil percentage, and lymphocyte percentage. All analyses were accounted for survey weights.
