# Supplementary material for: Aging measures and cancer in the Health and Retirement Study (HRS)
Source: Nat Commun. 2025 Jul 1;16:5916. doi: 10.1038/s41467-025-60913-z (PMC12215566; doi:10.1038/s41467-025-60913-z)
Supplement: Supplementary file 3 — Reporting Summary [file 41467_2025_60913_MOESM3_ESM.pdf]

nature portfolio

Corresponding author(s): Anna Prizment

Last updated by author(s): Apr 27, 2025

Reporting Summary

Nature Portfolio wishes to improve the reproducibility of the work that we publish. This form provides structure for consistency and transparency in reporting. For further information on Nature Portfolio policies, see our Editorial Policies and the Editorial Policy Checklist.

Statistics

For all statistical analyses, confirm that the following items are present in the figure legend, table legend, main text, or Methods section.

n/a	Confirmed	
☐	☒	The exact sample size (*n*) for each experimental group/condition, given as a discrete number and unit of measurement
☐	☒	A statement on whether measurements were taken from distinct samples or whether the same sample was measured repeatedly
☐	☒	The statistical test(s) used AND whether they are one- or two-sided<br>*Only common tests should be described solely by name; describe more complex techniques in the Methods section.*
☐	☒	A description of all covariates tested
☐	☒	A description of any assumptions or corrections, such as tests of normality and adjustment for multiple comparisons
☐	☒	A full description of the statistical parameters including central tendency (e.g. means) or other basic estimates (e.g. regression coefficient) AND variation (e.g. standard deviation) or associated estimates of uncertainty (e.g. confidence intervals)
☐	☒	For null hypothesis testing, the test statistic (e.g. *F*, *t*, *r*) with confidence intervals, effect sizes, degrees of freedom and *P* value noted<br>*Give P values as exact values whenever suitable.*
☒	☐	For Bayesian analysis, information on the choice of priors and Markov chain Monte Carlo settings
☒	☐	For hierarchical and complex designs, identification of the appropriate level for tests and full reporting of outcomes
☐	☒	Estimates of effect sizes (e.g. Cohen's *d*, Pearson's *r*), indicating how they were calculated

Our web collection on statistics for biologists contains articles on many of the points above.

Software and code

Policy information about availability of computer code

Data collection	No software was used. We used the publicly accessiable health and reitrement study (HRS) data.
Data analysis	We used the SAS version 9.4. We used proc surveymeans, proc surveyfreq, proc surveylogistic, and proc surveyphreg to perform the analyses in this study. The code used in the analysis can be accessed at https://github.com/wang8310/Aging_Cancer_HRS.

For manuscripts utilizing custom algorithms or software that are central to the research but not yet described in published literature, software must be made available to editors and reviewers. We strongly encourage code deposition in a community repository (e.g. GitHub). See the Nature Portfolio guidelines for submitting code & software for further information.

Data

Policy information about availability of data

All manuscripts must include a data availability statement. This statement should provide the following information, where applicable:

- Accession codes, unique identifiers, or web links for publicly available datasets
- A description of any restrictions on data availability
- For clinical datasets or third party data, please ensure that the statement adheres to our policy

All HRS data are publicly available on the HRS website (https://hrs.isr.umich.edu/data-products/).

Research involving human participants, their data, or biological material

Policy information about studies with human participants or human data. See also policy information about sex, gender (identity/presentation), and sexual orientation and race, ethnicity and racism.

Reporting on sex and gender	In the Health and Retirement Study (HRS), participants reported their sex at each survey. In this study, we reported the distributions of sex. Additionally, we adjusted for sex (female/male) in the model. We also examined the associations stratified by gender and compared the associations in females and males.
Reporting on race, ethnicity, or other socially relevant groupings	In the Health and Retirement Study (HRS), participants reported their race/ethnicity (Non-Hispanic White, Non-Hispanic Black, Hispanic White, Hispanic Black, or Other). In this study, we reported the distribution of race/ethnicity. Additionally, we adjusted for race/ethnicity in the model.
Population characteristics	In this study, we reported the distribution of participants' demographic and lifestyle characteristics, namely age, sex (female/male), race/ethnicity (Non-Hispanic White, Non-Hispanic Black, Hispanic White, Hispanic Black, or Other), body mass index (BMI), smoking status (self-reported; current, former, or never smokers), alcohol intake (ever drinking/never drinking), and their physical activity level. We also created a comorbidity index using seven self-reported conditions diagnosed by a physician. These conditions include hypertension, lung disease, cardiac disorders, stroke, arthritis, diabetes, and psychiatric problems. Moreover, we reported the distribution of cytomegalovirus (CMV) seroprevalence and muscle strength.
Recruitment	In this study, we investigated subjective age, clinical marker-based aging measures, and DNA methylation-based aging mearuses. In this Health and Retirement Study (HRS), clinical markers and DNA methylation markers were measured in different group of participants, so we resulted in two samples in this study: Sample A and Sample B. Sample A includes participants who reported their subject age and had biomarker measures used to calculate clinical marker-based aging measure in 2016. Sample B includes participants who had DNA methylation measures in 2016.
Ethics oversight	This study was approved by University of Minnesota Institutional Review Board. The studies were conducted in accordance with the local legislation and institutional requirements.

Note that full information on the approval of the study protocol must also be provided in the manuscript.

Field-specific reporting

Please select the one below that is the best fit for your research. If you are not sure, read the appropriate sections before making your selection.

[x] Life sciences [ ] Behavioural & social sciences [ ] Ecological, evolutionary & environmental sciences

For a reference copy of the document with all sections, see nature.com/documents/nr-reporting-summary-flat.pdf

Life sciences study design

All studies must disclose on these points even when the disclosure is negative.

Sample size	We included all eligible participants from the publicly accessible Health and Retirement Study (HRS). Our sample size was similar to or larger compared to previously published papers who examined similar associations, which suggested we have enough sample size to examine the associations.
Data exclusions	In our study, we excluded participants who did not report their cancer status, those who did not report their demographic, lifestyle, and comorbidity characteristics of interest (see above, population characteristics), and those without CMV measure (so-called eligible participants). In our study, we have two samples: Sample A and Sample B (see above). In Sample A, we studies aging measures, i.e., clinical-markers based aging measures and subjective age. In Sample B, we studies DNA methylation based aging measures. To create Sample A, among the eligible participants, we further excluded those who did not report their subjective age and those without data used to construct clinical marker based aging measures. To create Sample B, among the eligible participants, we further excluded those without DNA methylation measures.
Replication	In this study, we examined the association between biomarkers of aging and cancer prevalence, mortality among cancer survivors and people without cancer, and cancer risk. We did not replicated our results because we use the publicly accessible data, including biomarker measures. Most of our results are comparable to previous studies that had the same aims. We have discussed the comparison of our results with the results from previous studies in the discussion.
Randomization	Randomization is not relevant to this study. In this study, we examined the associations either in the whole sample or in cancer survivors and cancer-free participants (i.e., controls), separately. We defined participants as cancer survivors and cancer-free participants (i.e., controls) based on their response for the history of cancer question on the survey.
Blinding	Blinding is not relevant to this study because we used the publicly accessible Health and Retirement Study (HRS) data. We did not collect the data ourselves.

Reporting for specific materials, systems and methods

We require information from authors about some types of materials, experimental systems and methods used in many studies. Here, indicate whether each material, system or method listed is relevant to your study. If you are not sure if a list item applies to your research, read the appropriate section before selecting a response.

Materials & experimental systems

n/a	Involved in the study
☒	☐ Antibodies
☒	☐ Eukaryotic cell lines
☒	☐ Palaeontology and archaeology
☒	☐ Animals and other organisms
☒	☐ Clinical data
☒	☐ Dual use research of concern
☒	☐ Plants

Methods

n/a	Involved in the study
☒	☐ ChIP-seq
☒	☐ Flow cytometry
☒	☐ MRI-based neuroimaging

Plants

Seed stocks	We did not use plants in this study.
Novel plant genotypes	We did not use plants in this study.
Authentication	We did not plants in this study.
