# Supplementary material for: Aging measures and cancer in the Health and Retirement Study (HRS)
Source: Nat Commun. 2025 Jul 1;16:5916. doi: 10.1038/s41467-025-60913-z (PMC12215566; doi:10.1038/s41467-025-60913-z)
Supplement: Supplementary file 4 — Description of Additional Supplementary Files [file 41467_2025_60913_MOESM4_ESM.pdf]

Description of Additional Supplementary Files

Supplementary Data 1

Description: Description of aging constructs

Supplementary Data 2

Description: Regression coefficients for all the covariates adjusted for in the analysis ofassociations between aging constructs and cancer prevalence in 2016; HRS

Supplementary Data 3

Description: Regression coefficients for all the covariates adjusted for in the analysis of associations between aging constructs and mortality in cancer survivors; HRS (2016-2020)

Supplementary Data 4

Description: Regression coefficients for all the covariates adjusted for in the analysis of associations between aging constructs and mortality in cancer-free participants (controls); HRS (2016-2020)
